# Stromal cells in breast cancer as a potential therapeutic target

**DOI:** 10.18632/oncotarget.25245

**Published:** 2018-05-04

**Authors:** Samantha S. Dykes, Veronica S. Hughes, Jennifer M. Wiggins, Henrietta O. Fasanya, Mai Tanaka, Dietmar Siemann

**Affiliations:** ^1^ Department of Radiation Oncology, University of Florida, Gainesville, Florida, USA

**Keywords:** breast cancer, microenvironment, stromal cells

## Abstract

Breast cancer in the United States is the second most commonly diagnosed cancer in women. About 1 in 8 women will develop invasive breast cancer over the course of her lifetime and breast cancer remains the second leading cause of cancer-related death. In pursuit of novel therapeutic strategies, researchers have examined the tumor microenvironment as a potential anti-cancer target. In addition to neoplastic cells, the tumor microenvironment is composed of several critical normal cell types, including fibroblasts, vascular and lymph endothelial cells, osteoclasts, adipocytes, and immune cells. These cells have important roles in healthy tissue stasis, which frequently are altered in tumors. Indeed, tumor-associated stromal cells often contribute to tumorigenesis, tumor progression, and metastasis. Consequently, these host cells may serve as a possible target in anti-tumor and anti-metastatic therapeutic strategies. Targeting the tumor associated host cells offers the benefit that such cells do not mutate and develop resistance in response to treatment, a major cause of failure in cancer therapeutics targeting neoplastic cells. This review discusses the role of host cells in the tumor microenvironment during tumorigenesis, progression, and metastasis, and provides an overview of recent developments in targeting these cell populations to enhance cancer therapy efficacy.

## INTRODUCTION

Breast cancer is the most frequently diagnosed cancer among women. It is estimated that in 2018, over 260,000 new cases will be diagnosed and over 40,000 deaths will be attributed to breast cancer in the United States alone [1]. While therapeutic advances such as radiation, surgery, chemotherapy, and endocrine therapy have successfully lowered breast cancer mortality, therapeutic resistance leading to recurrence and/or metastasis is common [2–4]. As such, breast cancer represents a significant public health burden and the development of novel therapeutics targeting breast cancer progression are urgently needed.

Ductal carcinomas are the most common breast carcinoma, accounting for approximately 80% of all breast malignancies [5]. Ductal malignancies follow a linear tumor progression model, beginning with epithelial hyperproliferation, and then progressing to ductal carcinoma *in situ* (DCIS). The transition from DCIS confined within the duct to invasive ductal carcinoma is a critical step in breast cancer progression that often leads to metastatic disease, which is associated with high mortality [6, 7]. Metastatic progression is the leading cause of breast cancer-associated deaths, so identifying the mechanisms that contribute to metastasis is essential for the design of novel therapeutics.

Stephen Paget's seed and soil hypothesis proposes that tumor cells (seeds) can only grow where there is fertile soil (microenvironment) [8]. Indeed, modern evidence suggests that the stromal cells found within the microenvironment greatly influence both breast cancer initiation and metastatic progression. In this review, we will highlight the role of various stromal cells in breast physiology and the potential to target such cells in breast cancer (Table 1).

**Table 1:** Key cell types, their function, and potential therapeutic targets in the primary and metastatic breast tumor microenvironment

Cell	Function	Potential therapeutic targets
Fibroblast	ECM synthesis; cell scaffolding	Activation; Inhibition of FAP and TGF-β Normalization; Inhibit DNMT1 and JAK signaling
Vascular endothelial	Provide support and stability for blood vessels	Angiogenesis; VEGF MAPK/ERK and PI3K/Akt pathways
Lymph endothelial	Circulate immune cells, antigens, and macromolecules	Lymphangiogenesis; VEGFR2/3, VEGFC/D, NRP2, and PlexinA1/D1
Adipocytes	Lipid storage	Lipolysis, browning, IL-6, PHRP
Osteoclast	Bone resorption	RANKL, TGF-β, SRC, RON kinase, CTSL, CTSK
Innate Immune Cells (Macrophage, Neutorphil, Dendritic Cell)	Primary host defense against pathogens	GM-CSF, Dendritic cell activation, TLR7
Adaptive Immune Cells (T and B cells, Natural Killer Cells)	Antibody/cell mediated immune response	PD-1, CTLA-4, CD25, CCR4, LAG-3, and TIM-3

## FIBROBLASTS

Fibroblasts are ubiquitous cells in the tumor microenvironment, often comprising the bulk of stromal cells present in the tumor. Fibroblasts are implicated in cancer initiation, progression, and therapeutic resistance [9]. Attempts to target this cell population have been challenging, but have yielded some promising results.

### Functions in normal tissue

In normal tissue, fibroblasts are largely involved in the synthesis of the extracellular matrix (ECM), providing the physical scaffolding for cells. Fibroblasts secrete fibronectin and Type I, III, and V collagen, components of fibrillar ECM [10, 11] and the basement membrane components, Type IV collagen and laminin [12]. Additionally, proteases secreted by fibroblasts are involved in ECM remodeling and turnover [12, 13].

### Activated fibroblasts

Fibroblasts exist in either a normal or an activated state distinguishable by cell morphology and secretome. Activation of fibroblasts may occur in several ways, often by factors secreted from injured tissues. Two major factors involved in fibroblast activation are transforming growth factor-β (TGF-β) and stromal cell-derived factor-1 (CXCL12/SDF-1) [14, 15]. Other factors such as platelet-derived growth factor α/β (PDGF α/β), basic fibroblast growth factor (b-FGF), and interleukin-6 (IL-6) also play a role in fibroblast activation [16–20]. Fibroblast activation is a common feature in tumors; in breast carcinomas 80% of cancer-associated fibroblasts (CAFs) were found to be in an activated state [21].

After resolution of the injury in normal healthy tissue, the number of activated fibroblasts in the area diminishes [10]. Whether this occurs through reversion to a normal state or through apoptosis is unknown [10]. However, unlike fibroblasts in normal tissues, CAFs remain perpetually activated.

It has been proposed that the genomic instability of CAFs is responsible for their activated phenotype and altered gene expression profile. However, conflicting evidence regarding the genomic stability of CAFs in breast cancer confounds the identification of CAFs within the tumor. Although several groups have identified genomic abnormalities within the mammary tumor stroma including altered DNA copy number, and allelic loss in the fibroblast population [22–24], CAF genomic stability is hotly debated, and other groups have found no evidence of genetic alterations in CAFs from human breast carcinoma [25, 26]. The conflicting results are likely due to the varying methodologies by which tissue specimens were processed [25]. Ascertaining CAF genomic instability would facilitate the identification of CAFs, help define activated stroma borders, and may even affect clinical response to treatment.

### Effects of CAFs on the tumor microenvironment

Once activated, fibroblasts alter in proliferation, morphology and secretion characteristics, including elevated secretion of ECM-degrading proteases such as matrix metalloproteases 2 and 9 (MMP2 and MMP9) [27]. An increase in ECM remodeling and degradation is suspected to lead to an increase in metastasis. Moreover, collagen secretion is increased, and the types of collagen are secreted in different proportions in comparison to normal fibroblasts [28]. Additionally, increased secretion of growth factors such as vascular endothelial growth factor (VEGF), hepatocyte growth factor (HGF), epidermal growth factor (EGF), insulin-like growth factor (IGF), nerve growth factor (NGF), and fibroblast growth factor 2 (FGF2) may stimulate proliferation in epithelial cells, affecting vasculature as well as cancer cells [29]. Furthermore, there is evidence that activated fibroblasts are able to modulate immune cells through factors such as monocyte chemoattractant protein-1 (MCP-1) and IL-1, leading to a pro-inflammatory microenvironment [30, 31]. Interestingly, CAF phenotypes and expression profiles vary between breast cancer subtypes [32–34]. For example, CAFs derived from human epidermal growth factor receptor 2 (HER2+) breast cancers have increased expression of pathways associated with integrin signaling and the actin cytoskeleton, which may contribute to the fibroblast-driven collective invasion of cancer cells [34, 35]. This particular expression profile of CAFs may play a part in the aggressiveness and increased rate of recurrence of HER2+ breast cancers [36].

### CAFs in tumor initiation, progression, and therapeutic resistance

CAFs are believed to influence the initiation and progression of tumors. For example, breast cells co-injected into mice in the presence of CAFs formed larger, more vascularized tumors than tumor cells injected with normal fibroblasts [37]. Furthermore, *in vivo* injection of non-invasive cells with CAFs resulted in a more invasive phenotype [38].

Resistance to therapeutics also may be augmented indirectly by CAFs, via an increase in interstitial pressure within the tumor, reducing the efficacy of drug delivery [39]. CAFs also are suspected to contribute to tamoxifen resistance in breast cancer cells [40]. CAFs secrete TGF-β and HGF, which are known to stimulate several signaling pathways generally involved in drug resistance in tumor cells [41].

### Identification of CAFs

Due to the contribution of fibroblasts to cancer progression, there have been several attempts to target this cell population. However, identifying CAFs has been challenging, due to a lack of reliable cell markers. Several markers of fibroblasts have been utilized, including but not limited to vimentin [42–44], alpha-smooth muscle actin [10, 45], fibroblast-activation protein (FAP) [46, 47], fibroblast-specific protein-1 (FSP1) [48], and prolyl 4-hydroxylase [37, 49, 50]. However, expression of these markers is highly heterogeneous as fibroblasts have differing gene expressions based on organ and age of host [12]. Furthermore, there is a lack of specificity for theses fibroblast markers. The absence of a specific marker makes identifying and targeting fibroblasts challenging.

### Targeting CAFs as a therapeutic strategy

Several approaches have been taken to target CAFs. One method has been to inhibit CAF activation, by targeting CAF-associated proteins such as FAP. Sibrotuzumab, a FAP-targeting antibody, was tested in phase II trials for the treatment of metastatic colorectal cancer. Unfortunately, this agent failed to demonstrate efficacy [51]. Another protein of interest is DNA methyltransferase 1 (DNMT1), which is also involved in CAF activation. Preliminary studies indicate that combined inhibition of DNMT1 and Janus kinase (JAK) signaling resulted in normalization of fibroblasts [52]. Agents that target growth factors involved in fibroblast functions also have been evaluated. Pirfenidone, an anti-fibrotic agent with multiple functions including anti-TGF-β activity, inhibited tumor growth and metastasis in a preclinical triple negative breast cancer (TNBC) model when combined with doxorubicin [53]. Pirfenidone's effects may be due to a normalization of the tumor microenvironment, through reduction of collagen and hyaluronan levels, which may allow increased blood perfusion and drug delivery [54]. While targeting CAFs has potential to improve therapeutic efficacy, more research is needed to better understand the regulation of fibroblasts within the tumor microenvironment.

## VASCULAR ENDOTHELIAL AND LYMPH ENDOTHELIAL CELLS

Endothelial cells regulate important functions such as the transfer of nutrients, oxygen and other metabolic byproducts between the bloodstream and tissues, the movement and adhesion of leukocytes in the bloodstream, and the pressure of blood flow in the tumor microenvironment [55, 56]. Vascular endothelial cells and lymph endothelial cells, line blood and lymphatic vessels respectively. The endothelium has a highly organized hierarchical structure and interacts with other stromal and non-stromal cells to provide support and stability for the blood vessels. In contrast to the normal vasculature, the tumor vasculature is characterized by abnormal sprouts, intercellular gaps, and no hierarchical arrangement [57]. Vascular and lymph endothelial cells in the tumor microenvironment communicate with tumor cells and other stromal cells to promote tumorigenesis and metastasis.

### Vascular endothelial cells

Angiogenesis, also known as neovascularization, serves critical functions including supplying cells with nutrients, oxygen, and removing metabolic waste [56]. Vascular endothelial cells that line the blood vessel secrete a number of molecules for autocrine and paracrine signaling and to maintain blood flow homeostasis [56, 58]. Endothelial cells directly interact with other stromal cells to help maintain vessel integrity. For example, pericytes surround capillary endothelial vessels to regulate blood flow [59, 60]. Endothelial cells also interact with the ECM and basement membrane through integrins and proteoglycans for mechanical and physical support [61, 62].

In tumors, vascular development cannot keep pace with the nutritional demands of the rapidly proliferating neoplastic cell population. Consequently, oxygen transport to tumor cells is limited, resulting in hypoxic tumor microenvironments [63, 64]. Hypoxia induces the expression of hypoxia inducible factor (HIF) which upregulates a number of oncogenes, associated with an aggressive neoplastic cell phenotype [65, 66]. VEGF is one of the genes upregulated by HIF [67–69]. The secreted VEGF ligands bind to and activate VEGF receptors (VEGFR) on endothelial cells which, in turn, activate a number of downstream signaling pathways including the mitogen-activated protein kinases and extracellular signal-regulated kinases (MAPK/ERK) and phosphatidylinositol-3 kinases (PI3K)/Akt pathways that regulate cell survival, cell cycle progression, cell growth, and angiogenesis [70, 71]. Hence, hypoxia activates the VEGF signaling pathway to promote vascular permeability, angiogenesis, and neovascularization [72–74]. Activation of the VEGF signaling axis is a pro-angiogenic associated with tumor progression and poor prognosis in breast cancer [75].

### Targeting vascular endothelial cells

A number of anti-angiogenic drugs have been discovered and tested. In particular, targeting the VEGF signaling pathway for therapeutic intervention has shown efficacy in certain tumor types. However there is no FDA approved VEGF targeting agent for breast cancer treatment. The mTOR inhibitor Everolimus (Afinitor) is not regarded as an inhibitor of VEGF, yet it does possess anti-angiogenic properties. This agent has been approved for HER2-/hormone receptor (HR) + breast cancers. Clinically, there is limited efficacy for vascular targeting agents as single agents [63] and their combination with conventional therapies may be required for efficacy in some breast cancer types [76–78].

The lack of efficacy of anti-angiogenic agents may in part be due to the ability of endothelial cells to adapt and acquire drug resistance [79, 80]. Although endothelial cells are less likely to become drug resistant than tumor cells [81, 82], there are data indicating that tumor-associated endothelial cells can upregulate resistance genes including multi drug resistance 1 (MDR1), CD90, CD105, as well as other pro-angiogenic growth factors [83–86]. Furthermore, such endothelial cells can have cytogenetic abnormalities including aneuploidy or abnormal centrosomes [87, 88], unlike normal endothelial cells. Some suggest that these endothelial cells are derived from tumors, or tumor stem cells, and inherit genetic instability [89, 90]. Consequently, these endothelial cells may be able to escape and acquire resistance to anti-angiogenic therapies. Nonetheless, further understanding of the drug mechanisms of action on the tumor vasculature may be particularly important in improving progression-free and overall survival.

### Lymph endothelial cells

Lymph vessels carry immune cells, antigens and other macromolecules from the vascular system to the circulatory system [91]. In the tumor microenvironment, the formation of lymph vessels contributes to tumor dissemination by increasing the number of entry sites for metastatic tumor cells [92]. Due to the reduction of perivascular cells (pericytes) and smooth muscle cells to line the endothelium, lymphatic vessels are more permeable than blood vessels [93, 94]. These loose junctions in lymphatic vessels allow tumor cells to enter the lymphatic circulation and disseminate to the lymph nodes and distant organs.

Lymph endothelial and tumor cell interactions promote the metastatic phenotype. Lymph endothelial cells secrete chemokines including chemokine (C-X-C motif) ligand 12 (CXCL12) and chemokine (C-C motif) ligand 21 (CCL21) to recruit tumor cells that express chemokine (C-C motif) receptor 7 (CCR7) and chemokine (C-X-C motif) receptor 4 (CXCR4) [95, 96]. Lymph endothelial cells also secrete C-C chemokine ligand 5 (CCL5), which increases the migratory capacity of TNBC cells that express C-C chemokine receptor 5 (CCR5) [97, 98].

### Targeting lymph endothelial cells

Currently, there are no clinical agents that selectively target lymph endothelial cells. Preclinical agents for anti-lymph angiogenesis target molecules such as VEGFR2/3, VEGF-C/D, neuropillin-2 (NRP2), and Plexin-A1/D1 [99–103]. These molecular targets are not specific for lymph angiogenesis, but also target blood vessel angiogenesis. Still, *in vitro* and *in vivo* preclinical studies of such agents demonstrate that inhibition of lymph angiogenesis reduces lymph node and distant metastases [98–100, 103]. This line of investigation may have particular relevance in inflammatory breast cancer (IBC), a rare form of aggressive breast cancer characterized by a rapid onset of erythema, rigidity, and edema of the skin of the breast [104, 105]. The signs and symptoms of IBC are often associated with the blockage of the lymphatic vasculature by a tumor emboli which is often misdiagnosed as mastitis or bacterial infection [106]. Lymphatic involvement is associated with a high rate of recurrence, so targeting lymph endothelial cells in IBC may alter disease progression [107].

## ADIPOCYTES

Adipocytes specialize in the storage and synthesis of lipids and classically were thought of as a long-term fuel reserve, which could be accessed during times of nutrient deprivation. However, recent evidence suggests that adipocytes are a complex cell population with varying roles in both homeostasis and cancer progression. Adipocytes can be classified roughly into two populations, lipid storing white adipocytes, and thermogenic brown adipocytes. White adipocytes are characterized morphologically as having a single lipid inclusion body that occupies the majority of the space within cytoplasm and is essential for lipid storage. In contrast, brown adipocytes contain an increased number of mitochondria and often have multiple smaller lipid droplets [108]. As adipocytes make up a large percentage of tissue (average of 48%) within the mammary fat pad, their contributions to normal breast physiology and to breast cancer are significant [109, 110].

### Role of adipocytes in homeostasis

In addition to lipid storage, white adipose cells are important players in in the production and secretion of hormones, most notably leptin and estrogen [111]. Adipose tissue plays a role in the development of the mammary duct formation and vasculature. In humans, the neonatal ductal structures have been found juxtaposed to vascularized embryonic adipose islands [112, 113]. In the mammary fat pad, adipose cells provide growth factors such as VEGF, which promotes mammary vascular development [114]. Mammary adipose tissue also produces and secretes IGF-I, which contributes to the growth of the ductal epithelium [115, 116]. Thus, mammary adipocytes may regulate angiogenesis and subsequent epithelial function through the secretion of growth factors and other soluble components. The mammary tissue continues to develop during puberty and during the pregnancy-lactation-involution transitions. During lactation, adipose tissue undergoes lipolysis to release lipids and metabolites in support of milk production [117–119]. During post-lactation involution, mammary adipocytes replenish their lipid stores in response to local ECM remodeling [120, 121].

### Cancer associated adipocytes and the role of adipocytes in breast cancer

Until recently, adipocytes were thought to be an inert cell population, but evidence suggests that adipocytes located in close proximity to the growing tumor take on a pro-tumorigenic phenotype, termed ‘cancer-associated adipocytes’ [122]. Co-culture experiments revealed that adipocytes co-cultured with breast cancer cells overexpress inflammatory cytokines such as IL-6, TNFα, and MMPs, which promote tumor cell invasion [123]. Additionally, co-cultured adipocytes exhibited a reduced lipid accumulation due to lipolysis. Fatty acids released from adipocytes, can be taken up by neighboring cancer cells [124]. These profound phenotypic changes suggest that adipocytes can become activated in response to tumor cell proximity and in turn, supply pro-tumorigenic factors that stimulate cancer cell invasion [122, 123].

While the role of cancer associated adipocytes in tumor progression is relatively novel, it is clear that altered adipocyte metabolism plays a role in cancer-associated cachexia, a wasting condition that negatively impacts both patient quality of life and disease prognosis. Cancer-associated cachexia is defined as a “multifactorial syndrome defined by an ongoing loss of skeletal muscle mass (with or without loss of fat mass)” [125]. Muscle wasting is a hallmark of cachexia and is a direct consequence of adipocyte lipolysis, which releases free fatty acids that can be taken up by skeletal muscle cells, resulting in muscle atrophy [126–128]. In addition to lipolysis, white-to-beige adipocyte trans-differentiation (browning) is associated with cancer-associated cachexia. Tumor-secreted factors such as IL-6 and parathyroid hormone-related peptide (PHRP) can stimulate browning, resulting in the increased energy expenditure of adipose tissue contributing to cancer-associated cachexia [129, 130].

### Cancer treatments targeting adipocytes

The contributions of adipocytes to breast cancer progression and cancer-associated cachexia are relatively young fields of study. It is likely that as the mechanisms regulating adipocyte-cancer cell crosstalk are elucidated, drug targets will be developed.

## BONE CELLS

### Bone homeostasis

The major functions of the skeletal system are calcium homeostasis, hematopoietic cell production, locomotion, support, and protection of internal organs [131]. The coordinated action of osteoblast and osteoclast are responsible for bone remodeling that occurs during normal skeletal maintenance. Osteoblasts synthesize and mineralize bone matrix, and osteoclasts are responsible for bone resorption. In calcium homeostasis, parathyroid hormone (PTH) is released in response to low calcium levels; this process activates the osteolytic release of calcium from the bone [131] by stimulating the expression of receptor activator of NF-kappa-β Ligand (RANKL) from osteoblasts [132, 133]. RANKL binds to RANK on immature osteoclasts, initiates osteoclastogenesis, and bone resorption. Recently, Andrade *et al*. demonstrated that RON tyrosine kinase mediated macrophage-stimulating protein (MSP) signaling by the host bone microenvironment activates osteoclasts in a complementary fashion to RANK-RANKL signaling [134].

Estrogen regulates skeletal development and homeostasis in women, through estrogen receptors found on bone cells. Estrogen represses the expression of critical osteoclastogenic factors such as IL-6, Tumor necrosis factor-alpha (TNF- α), and RANKL [135, 136], resulting in osteoclast apoptosis [137, 138].

### Clinical course of bone metastases in breast cancer

The bone is the most common site of distant metastasis for all molecular subtypes except basal-like tumors [139]. Bone metastasis account for ~70% of all breast cancer metastasis [140]. The five year survival of patients with metastatic breast cancer is 26% [141], and bone metastasis development is correlated with high morbidity and mortality [142, 143].

The most common sites of breast cancer bone metastases are the spine, rib and sternum, and the proximal ends of long bones [144]. Patients with bone metastasis experience skeletal related events (SREs) such as pain, hypercalcemia, pathologic fractures, and spinal cord compressions [140, 145]. Breast cancer bone metastases are primarily osteolytic (~80%), contribute to SREs, and significantly affect overall quality of life.

Breast cancer cells express osteo-specific factors, to increase survival within the bone environment through osteomimicry [146, 147]. Osteotropic breast cancers overexpress bone mineralization and differentiation proteins such as Runt-related transcription factor 2 (RUNX2), bone sialoprotein II, osteoactivin, ectoenzyme ectonucleotide pyrophosphatase 1 (ENPP1) and adrenomedullin (AM) [146, 148, 149]. By expressing these osteotropic proteins, breast cancer cells begin to colonize and establish themselves in the bone.

The interaction of breast cancer cells and bone stromal cells initiate a vicious cycle, in which tumor cells secrete factors that facilitate bone remodeling. Concurrently, the release of growth factors from the nutrient rich bone environment provides a hospitable environment for tumor cells. Degradation of the bone matrix releases growth factors such as TGF-β, FGF and bone morphogenetic proteins (BMPs), which provide fertile ground for breast cancer cells to grow [143, 146, 147]. In normal mammary epithelial cells TGF-β is a negative growth regulator, however TGF-β promotes tumor development within the bone environment [150]. Breast cancer cells colonize the bone by binding to vascular cell adhesion proteins (VCAM1), N-cadherin, RANKL [149]. Inflammatory cytokines such as IL-1, IL-6, IL-8, and IL-11 increase osteoclast activation and bone resorption through TGF-β and RANKL expression [131, 142, 150–152]. Several studies have highlighted the importance of chemokine binding through the CXCR4-CXCL12 axis in breast cancer and metastasis [96, 153–156]. CXCR4 is elevated in malignant breast tissue and bone metastasis [153]. Src protein tyrosine kinase is activated in osteoclast, suggesting that Src plays a pivotal role in bone remodeling [157]. Furthermore, Src activation has been correlated to CXCR4-CXCL12 signaling and bone metastasis in breast cancer [158].

In breast cancer, cells secrete PTH related peptide, which activates osteoclasts leading to the degradation of the bone matrix and the release of pro-tumorigenic growth factors [142, 151, 159]. Osteolytic bone resorption is facilitated by the release of proteases that degrade the bone matrix. Cathepsin K (CTSK), is a protease that is secreted from both metastatic breast cancer cells and osteoclasts to promote degradation of the bone matrix [160]. The hyperactive bone resorption by osteoclasts causes extracellular calcium levels to rise up to 40 fold, leading to hypercalcemia [161].

### Traditional therapeutics

Currently, FDA approved osteoclast-targeted agents such as bisphosphonates, which induce osteoclast apoptosis, and anti-RANKL antibodies, which block osteoclast formation, specifically zoledronic acid and desonusmab respectively, are used to treat patients with bone-related metastatic breast cancer [162]. Although evidence suggests that osteoclast-targeted agents decrease bone remodeling and pain, these therapeutics do not increase overall survival of patients with metastatic breast cancer. Therefore, cytotoxic or cytostatic agents in combination with osteoclast-targeted agents may provide an increase in overall survival, as well as improved quality of life. As we begin to understand the molecular mechanisms underlying bone metastases, new therapies that target bone remodeling pathways hold great promise.

### Potential new therapies

Strategies to reduce the incidence and morbidity of bone metastasis are of great clinical importance. Targeting molecular pathways that regulate the bone metastasis microenvironment may be beneficial to decreasing morbidity and mortality in patients with advanced breast cancer. Inhibitors of TGF-β may decrease osteolytic metastasis by interrupting the vicious cycle [163]. Preclinical studies demonstrate that Src inhibitors decrease bone metastases and improved survival of tumor bearing mice [157]. Recently, RON kinase and Cathepsin K inhibitors have demonstrated a reduction in breast cancer induced osteolysis and skeletal tumor burden in preclinical and clinical studies [134, 160, 164]. Targeting Cathepsin L (CTSL), whose upregulation is associated with poor clinical outcomes of breast cancer patients, may offer another approach that could be of significant benefit in the treatment of metastatic cancer patients. Preclinical studies have reported that pharmacological inhibition of CTSL significantly impairs the invasive and metastatic capacities of human breast and prostate cancer cells through suppression of pro-angiogenic and bone remodeling functions of tumor cells [165, 166]. Further understanding of the tumor-bone interaction may identify the best potential targets for chemotherapeutic and microenvironmental-targeted agents to halt bone metastasis, reduce the incidence of SREs and improve overall survival. Innate Immune Cells.

### Innate immune cells in mammary development

The innate immune system consists of eosinophils, basophils, mast cells, natural killer cells, phagocytic macrophages, dendritic cells, and neutrophils [167]. Together, these cells function as a non-specific first line of defense against foreign antigens and activate the adaptive immune response [167]. Innate immune cells regulate multiple aspects of breast development as the mammary tissue continues to change during the postnatal period and with every pregnancy [168]. In normal breast development, eosinophils and macrophages play a key role in regulating branching morphogenesis and ductal outgrowth [169]. During post-lactation involution, milk stasis triggers the apoptosis of mammary epithelial cells [170]. Macrophages, neutrophils, and eosinophils are recruited during mammary tissue involution to clear residual milk and cellular debris [171–173]. Additionally, innate immune cells are thought to supply many of the proteases, including MMPs, that contribute to ECM remodeling during the involution process [174]. The immune response that occurs during mammary tissue involution is carefully coordinated to clear and remodel the breast tissue without triggering a massive systemic immune response. In addition to mammary development, the natural killer cells of the innate immune system are critically involved in immunosurveillance to suppress the outgrowth of cancer cells [175].

### Innate immune cells in breast cancer

While immunosuppression is essential for the normal functioning breast tissue, these mechanisms may be hijacked to promote an environment that is favorable for tumor growth. For example, the innate immune-mediated wound healing that occurs during involution is associated with an increased risk of breast tumor development [176]. The link between inflammation and cancer development is clear and the innate immune system can contribute to both breast cancer development and progression. Due to their therapeutic potential, this section will focus on the role of macrophages, myeloid-derived suppressor cells, neutrophils, and dendritic cells in breast cancer development.

Notably, the presence of tumor-associated macrophages (TAMs) within the breast tumor correlates with poor prognosis [177–179]. Breast cancer cells recruit macrophages via the production of macrophage colony stimulating factor (MCSF) and stimulate macrophages to take on an immunosuppressive or M2 phenotype via the production of IL-4 [180–182]. TAMs suppress the anti-tumor immune response via the secretion of anti-inflammatory cytokines, including IL-10, and supply the tumor with growth factors such as EGF and VEGF, which promote primary tumor growth and angiogenesis [179, 183, 184]. In addition, TAMs contribute to tumor cell invasion by secreting proteases, including lysosomal cathepsins and MMPs, which degrade the ECM [182, 185].

Myeloid-derived suppressor cells (MDSCs) are a heterogeneous population of immature myeloid lineage cells that can be found within the breast tumor and peripheral lymph nodes [186]. MDSCs function primarily as suppressors of the innate and adaptive immune system, but can also promote tumor growth and metastasis. Under conditions of chronic inflammation, such as cancer, MDSCs are released from the bone marrow and undergo continuous expansion in response to tumor secreted-growth factors such as MCSF, VEGF, TNF-α, and IL-6 [187, 188]. In the local tumor microenvironment, MDSCs suppress the anti-tumor adaptive immune response through various mechanisms. MDSCs upregulate the expression of the L-arginine converting enzymes, inducible nitric oxide synthase (iNOS) and arginase-1 (ARG-1), which convert L-arginine to into nitric oxide and urea and L-ornithine, respectively [189]. L-arginine is essential for normal T-cell function, so the metabolism of L-arginine by MDSCs suppresses T-cell function [190]. In addition to their immunosuppressive role, MDSCs are thought to promote breast cancer metastasis by priming the pre-metastatic niche and facilitating tumor cell mesenchymal to epithelial transition [191, 192].

Although controversial, mounting evidence suggests that neutrophils also promote breast cancer growth and metastatic progression. Indeed, tumor cells secrete IL-8, a neutrophil chemoattractant, suggesting that neutrophils are not passively infiltrating tumor tissue [193]. Additionally, *in vivo* studies revealed that neutrophil depletion slows primary tumor growth and reduces the number of breast cancer metastasis [194, 195]. Several mechanisms contributing to the role of neutrophils in cancer have been proposed. Neutrophils supply matrix metalloproteinases that can remodel the ECM and promote tumor cell invasion [196]. Furthermore, neutrophils promote early tumor angiogenesis via the proteolytic activation of VEGF [197]. The number of infiltrating neutrophils varies with each breast cancer subtype, confounding the overall contributions of neutrophils in breast cancer [198].

Dendritic cells represent the link between the innate and adaptive immune response, whereby dendritic cells survey the microenvironment and present antigens to T cells. Dendritic cell infiltration into breast carcinoma is inversely correlated with tumor grade and prognosis [199]. However, tumors can reduce dendritic cell function and numbers as a means of immune evasion. Tumors cells can induce the apoptosis of infiltrating dendritic cells, thereby preventing effective tumor antigen presentation [200, 201]. Additionally, the presence of IL-6 within the tumor microenvironment can promote dendritic cell to macrophage differentiation, thus suppressing the activation of the anti-tumor adaptive immune response [202]. Often, tumor infiltrating dendritic cells are functionally deficient which, in part, contributes to immune tolerance [203].

### Therapeutic targeting of the innate immune system

The innate immune system contains many diverse cell populations, many of which contribute to breast cancer progression. While the innate immune system represents an attractive target for the treatment of breast cancer, there are no FDA approved immunotherapies for the treatment of breast cancer, but many agents are in early phase clinical trials. Several treatment strategies aimed at modulating the innate immune system are discussed below.

Due to their ability to activate the adaptive immune response, dendritic cells are the targets of many therapeutic interventions. Various iterations of dendritic cell vaccines are in clinical trials for the treatment of breast cancer. Recently, a HER2 peptide-pulsed dendritic cell vaccine was used to treat HER2+ ductal carcinoma *in situ* and early invasive breast cancer [204]. The dendritic cell vaccine was well tolerated and adequately mounted an anti-HER2 immune response in participants with ductal carcinoma *in situ*. These studies suggest that dendritic cell vaccines may be more effective in the treatment of early stage breast cancers [204].

IRX-2 is a defined mixture of cytokines that is used to promote dendritic cell function and antigen presentation. Administration of IRX-2 was found to promote tumor specific antigen presentation and overall survival in head and neck squamous cell carcinoma patients in phase II clinical trials [205]. While IRX-2 is not currently FDA approved, a phase I clinical trial to study the effects of IRX-2 administration in pre-operative early stage breast cancer is currently recruiting patients.

Treatment of granulocyte-macrophage colony-stimulating factor (GM-CSF) activates innate immune cells and increases the expression of tumor-associated antigens on tumor cells. Several ongoing studies are investigating the efficacy of GM-CSF (Sargramostim)/trastuzumab (anti-HER2 antibody) combination therapy for the treatment of HER2+ breast cancer [206]. Sargramostim is approved for the treatment of neutropenia and to reconstitute the myeloid lineage cells following chemotherapy and transplant. There is hope that the Sargramostim-driven proliferation of myeloid lineage cells will also help the body's immune system mount an anti-tumor immune response in combination with standard of care treatment.

Yet another innate immune-targeting agent is the toll-like receptor-7 (TLR7) agonist, 852A. Toll-like receptors are expressed preferentially in innate immune cells [207], and their activation promotes crosstalk between the innate and adaptive immune system, which is essential for a potent anti-tumor response [208]. Treatment with 852A induced immune activation in breast cancer patients, but prolonged treatment resulted in cardiotoxicity [209].

Immunotherapy for the treatment of breast cancer is still a relatively young field. The diversity and fluidity of the innate immune system makes harnessing it for targeted therapeutics a difficult task and many innate immune targeting agents are still in clinical development. Further investigation is warranted to understand the mechanisms governing the innate immune response in breast cancer.

## ADAPTIVE IMMUNE SYSTEM

### The adaptive immune response

The adaptive immune system, also known as the acquired immune response, is a late stage immune response generally involving either an antibody-mediated/humoral or a cell-mediated response [210]. The main players in this response are derived from the lymphoid cell lineage, which is composed of T-cells and B-cells. Natural Killer cells (NK cells), also nascent from the lymphoid lineage, are not directly involved in the adaptive response. However, in order to enact a full adaptive response, both lymphoid and myeloid cells collaborate to eliminate foreign pathogens or in this case, tumor cells.

T-cells (thymus derived) can be divided into several cell subtypes, broadly: those expressing the CD8+ glycoprotein, such as Cytotoxic T-cells (CTCs); or those expressing the CD4+ glycoprotein, T-helper (Th) cells, T-follicular helper (Tfh) cells, and T-regulatory cells (T-regs) [211]. B-cells (*b*one marrow derived) are a group of cells that terminally differentiate into plasma cells, which clonally express antibodies or immunoglobulin (Ig) receptors targeting “non-self’ or aberrant antigens present on the surface of abnormal cells [212].

An antigen is defined as a signaling molecule that triggers the adaptive immune response, in other words an *anti*body *gen*erator [210]. In particular, cancer cells will express antigens unique to tumor cells in addition to normal antigens categorized as “self” specific to the tissue from which they are derived. These tumor specific antigens can be classified as: oncofetal (exclusive to fetal development), oncoviral (derived from oncolytic viruses), mutated genes, overexpressed genes, lineage restricted (from a specific lineage), male germline restricted (thus not part of the “self” repertoire); post-translationally altered or idiotypic (polymorphic genes expressed clonally within a tumor) [213, 214].

### The adaptive immune response to cancer

The presence of antigens on tumor cells can trigger the activation of the humoral immune response. Briefly, it can be described as the plasma cell release of tumor-antigen specific antibodies that can block receptor interactions, tag tumor cells for phagocytosis or activate the complement cascade [215]. Alternatively, pathogens and tumor cells can also be targeted by the cell-mediated immune response. Here, antigen presenting cells (APCs) will express aberrant antigens on their major histocompatibility complex (MHC) I or II, which recognize and bind to the T-cell receptor (TCR) to activate Th cells or CD8+ T cells. Importantly, full activation of CD8+ T cells requires the co-stimulation complex CD28/B7-molecule to enable the release of cytotoxic cytokines upon encounter with the antigen [215].

The immune system hence is armed with the appropriate tools to target and eliminate abnormal and neoplastic cells. Indeed, normal cells are constantly under attack by endogenous and exogenous factors which generate over 10^4^ DNA lesions a day [216] causing oncogenic or tumor suppressor mutations. If not corrected by the DNA damage repair machinery the resulting peptides from these mutations are naturally exposed on the cell surface of damaged cells, which then are recognized by APCs. Thus by immunological surveillance, the immune system is capable of eliminating these aberrant cells and avoid the formation of cancerous lesions [217].

Tumor cells avoid detection and destruction by the immune systems in many ways. Some of these mechanisms involve avoiding antigen presentation, upregulating T-regulatory cell activity, suppressing immune mediators or downregulation of co-stimulatory molecules [218]. Briefly, cancer cells are able to downregulate the expression of the major histocompatibility complex (MHC) class I receptors on their cell surface thereby becoming invisible to the immune system [219]. Additionally, T-regulatory cells, which are responsible for maintaining immune homeostasis, are recruited by cancer cells to the tumor mass where they expand and promote an immune suppressive microenvironment [220]. Specifically, T-regs inhibit CD8+ T cells to suppress their activity and activate their apoptotic pathway most notoriously through the interaction of PD-1 (programmed cell death protein 1) with its ligand PD-L1 on the surface of T-regulatory cells. However, T-regs also possess a number of other upregulated receptors involved in T-cell suppression, such as the cytotoxic T-lymphocyte antigen 4 (CTLA-4) [221] that binds to B7 molecule on T-cells and APCs thereby inhibiting the co-stimulation of these cells. Furthermore, T-regs can also abrogate B-cell function further inhibiting the adaptive immune response [221].

### Therapeutic targeting of the adaptive immune system

The vast majority of immunotherapies to date are focused on enhancing the adaptive immune system. Current preclinical and early phase clinical trial treatment modalities include adoptive cell transfer, therapeutic antibodies, cancer treatment vaccines, system modulators, and immune checkpoint modulators. The latter are of particular interest of late. The goal of therapeutics targeting checkpoints such as PD-1, CTLA-4, CD25 inhibitors, among others currently in clinical trials (CCR4, LAG-3, TIM-3, etc.), is to disrupt this interaction and blunt T-regulatory cell activity [220].

Breast cancer typically does not possess a large number of infiltrating immune cells [222]. However there is evidence that the fraction of infiltrating immune cells, and more specifically lymphocytes, are associated with better prognosis in TNBC [222] as well as estrogen and progesterone receptor positive (ER+; PR+) breast cancer subtypes [223]. Interestingly, the presence of tumor infiltrating lymphocytes is associated with adverse prognosis for survival in HER2- luminal breast cancer. Additionally, recent evidence suggests that TNFα, IL-6, and TGFβ secreted from TCR-activated T cells induces EMT in inflammatory breast cancer cells [224]. This suggests that different breast cancer subtypes have varying immunological infiltrates and that immune modulating therapies should be tailored to cancer subtype [225]. Although none of these studies showed a positive correlation with HER2+ breast cancer subtypes, perhaps due to the small sample size, recent evidence suggests the T-cell signature may be more predictive than just the number itself. Indeed, Bense *et al*. observed that a higher fraction of γδ T-cells, a lesser known type of CD8+ T-cell, correlated positively with improved overall survival in all breast cancer subtypes, including HER2+ breast cancer patients [226]. Conversely, a higher proportion of T-regulatory cells was associated with poor prognosis in the HER2+ breast cancer subtypes [226].

Immune checkpoint inhibitors have been applied successfully in some cancers, most notably melanoma [227]. Clinical trials in breast cancer are ongoing. Such trials have focused primarily on TNBC, which has shown a higher expression of PD-L1 than hormone receptor positive tumors that correlated with the fraction of infiltrated CD8+ T-cells [228]. Pembrolizumab, a PD-1 inhibitor recently has been tested in TNBC patients, who tested positive for PD-L1 expression (58.6% of TNBC patients tested), in a phase1b clinical trial where only 15% of patients experienced adverse reactions to the drug and 37.5% showed a decrease in tumor diameter [229].

Other ongoing therapies include adoptive cell transfer, where peripheral blood is taken from patients and naïve immune cells are differentiated and specialized to target tumor cells, expanded *in vitro* and then reinfused into the patient. One such study currently recruiting patients in China (NCT03183206) is focused on adoptive cell transfer of γδ T-cells in cohorts of breast cancer patients, regardless of their hormonal subtype. Although no results have been posted to date, this method may hold promise in many cancer settings, including breast cancer, due to the ability of γδ T-cells to selectively target tumor cells. The ease with which they can be genetically modified and their low reactivity to the immune system, also makes them prime candidates for both autografts and allografts [230].

CAR-T cells are engineered T-cells that have been genetically modified to express a chimeric antigen receptor (CAR) specifically targeting tumor cells [231]. Recently, the FDA approved the use of CAR-T cell therapy for a form of acute lymphoblastic leukemia and advanced lymphoma, making this form of personalized medicine the first of its kind [232]. There are very few clinical trials using CAR-T cell therapy. One, based at the Fred Hutchinson Cancer Research Center (NCT0206392), is targeting TNBC cells, as well as other malignancies, with a modified receptor tyrosine kinase-like orphan receptor 1 positive (ROR1+) CAR-specific T-cell, which is expressed in 22% of TNBC patients [233]. Although this therapy is still in its infancy, it holds promise in many cancer settings.

Furthermore, recent approaches seek to improve immune detection of breast cancer cells by administration of cancer vaccines. One study focused on Globo-H, a cancer-associated carbohydrate that is expressed in a large number of breast cancers but is poorly detected by the immune system (NCT01516307). Metastatic breast cancer patients were subject to a combination therapy of the Globo-H-KLH vaccine and a low dose of cyclophosphamide or cyclophosphamide and saline as control. Results showed a significant increase in progression free survival and overall survival exclusively in the group of patients with high IgG titers after vaccine administration. While further research is needed to elucidate the mechanism underlying the differences between responders and non-responders, this approach illustrates a means to target tumors with lower immunogenicity.

## CONCLUSIONS AND PERSPECTIVES

Breast cancer remains a major health concern. While significant improvements in breast cancer managements have been made, treatment resistance and cancer cell dissemination limit the success of current therapeutic strategies. Novel agents designed to target invasive and metastatic breast carcinoma are urgently needed.

While most therapeutic strategies have focused on improving tumor cell kill, breast tumors consist of multiple stromal cell types in addition to the neoplastic cells. These host cells, fibroblasts, adipocytes, bone cells, immune cells, vascular and lymph endothelial cells, all can contribute to breast cancer progression and dissemination through a variety of mechanisms. Thus targeting them may provide an effective means to enhancing anti-tumor efficacy. Furthermore, the relative genomic stability of stromal cells make them ideal targets for novel anti-cancer therapeutics, as the risk of evolving therapeutic resistance is low. While only a few stromal-targeted therapeutics are approved for breast cancer treatment, many agents are currently in pre-clinical development and early phase clinical trials. Still, it is likely that many stromal-targeted agents will enter the clinical arena in the future.

While stromal-targeted therapy is appealing, there are some potential clinical concerns that need be considered. Most critically, will such an approach be selective, or will stromal-targeted therapies have deleterious effects on non-cancerous tissue homeostasis? To date, the identification of cancer-activated stromal phenotypes suggest that the neoplastic and normal stroma differ significantly; an encouraging observation. Another concern is that the tumor stroma may evolve as tumors progress. This would require different stromal-targeted interventions at different stages of disease progression. In addition, evolving tumor stroma could lead to the development of resistance to stromal-targeted therapy. Furthermore, there is mounting evidence that the tumor stroma may differ between breast cancer subtypes. Current treatment designs depend heavily on breast cancer molecular subtype, and the contribution of unique subtype-associated stromal cells may influence therapeutic outcome. These considerations coupled with a clearer understanding of the molecular mechanisms that drive cancer-associated stromal activation are necessary to design safe and effective stromal-targeting agents.

Finally, it is unlikely that stromal-targeting agents on their own will be curative. Rather such agents will have their greatest utility when coupled with conventional breast cancer-targeting therapeutics. Rigorous clinical trial design and adequate pre- and post-treatment sample acquisition are needed to help form firm conclusions about the benefits of stromal-targeting agents.

## Abbreviations

APC: antigen presenting cell
CAF: cancer-associated fibroblast
CAR-T cell: chimeric antigen receptor T-cell
CTLA-4: cytotoxic T-lymphocyte antigen 4
CD8+: cytotoxic T-cells
DCIS: ductal carcinoma *in situ*
DNMT1: DNA methyltransferase 1
ECM: extracellular matrix
EGF: epidermal growth factor
FGF: fibroblast growth factor
HER2: human epidermal growth factor receptor 2
HGF: hepatocyte growth factor
HIF: hypoxia inducible factor
HR: hormone receptor
IGF: insulin-like growth factor
IL: Interleukin
MCP-1: monocyte chemoattractant protein-1
mCSF: macrophage colony stimulating factor
MDSC: myeloid-derived suppressor cells
MHC: major histocompatibility complex
MMP: matrix metalloprotease
PD1: programmed cell death protein 1
PDGF: platelet-derived growth factor
PTH: parathyroid hormone
RANKL: receptor activator of NF-kappa-β ligand
SRE: skeletal related event
T-regs: T regulatory cells
TAM: tumor-associated macrophage
TCR: T-cell receptor
TGF-β: transforming growth factor-beta
TNBC: triple negative breast cancer
TNFα: tumor necrosis factor α
VEGF: vascular endothelial growth factor
